# Diagnostic Performance of 60 MHz High-Definition Intravascular Ultrasound versus Fourier Domain Optical Coherence Tomography for Identifying Plaque Rupture, Plaque Erosion, and Thrombosis in a Rabbit Model

**DOI:** 10.31083/j.rcm2403076

**Published:** 2023-03-03

**Authors:** Gang Wang, Weishen Qiao, Chun Xing, Zhibo Yao, Yufei Sun, Xingtao Huang, Xuedong Wang, Qi Liu, Ruoxi Zhang, Xing Luo, Yongmei Yu, Jiannan Dai, Jingbo Hou, Bo Yu

**Affiliations:** ^1^Department of Cardiology, The Second Affiliated Hospital of Harbin Medical University, 150000 Harbin, Heilongjiang, China; ^2^Key Laboratory of Myocardial Ischemia, Chinese Ministry of Education, 150000 Harbin, Heilongjiang, China; ^3^Department of Cardiology, Harbin Yinghua Hospital, 150000 Harbin, Heilongjiang, China

**Keywords:** acute coronary syndrome, 60 MHz HD-IVUS, animal model, hemodynamic flow, atherosclerotic plaque morphology, plaque rupture, plaque erosion

## Abstract

**Background:**

Most acute coronary syndromes occur due to coronary thrombosis caused by plaque rupture (PR) and plaque erosion (PE). Precise *in vivo* differentiation between PR and PE is challenging for intravascular imaging. This study is the first to determine the diagnostic performance of the novel 60 MHz high-definition intravascular ultrasound (HD-IVUS) for differentiating atherosclerotic plaque morphology influenced by local hemodynamic flow in rabbits. This study evaluated the diagnostic performance of 60 MHz HD-IVUS in identifying thrombosis in rabbits.

**Methods:**

We established 60 rabbit models of atherosclerosis with left common carotid artery (LCCA) stenosis and 30 ${\mathrm{FeCl}}_{3}$-induced LCCA thrombosis. Intravascular imaging was assessed with 60 MHz HD-IVUS and fourier-domain optical coherence tomography (FD-OCT). The present study investigated the diagnostic accuracy of 60 MHz HD-IVUS for PR and PE, as well as thrombosis, using OCT-diagnosis as a standard reference.

**Results:**

60 MHz HD-IVUS for identifying atherosclerotic plaque morphology using plaque cavity and minor intimal irregularities showed high sensitivity and specificity; 92.0 and 90.0% for identifying OCT-defined PR, and 80.0 and 70.0% for OCT-defined PE, respectively. In a rabbit thrombus model, 60 MHz HD-IVUS showed high sensitivity (88.0%) and specificity (80.0%) in identifying OCT-defined thrombosis.

**Conclusions:**

60 MHz HD-IVUS can accurately identify PR and thrombosis. Further studies should confirm the clinical value of this novel technique in PE diagnosis.

## 1. Introduction

Acute coronary syndrome (ACS) develops as a result of a sudden decrease in myocardial blood flow after thrombosis. Pathologically, plaque rupture (PR) accompanied by acute thrombosis is the primary mechanism, accounting for 67% of all ACS cases. The second most common underlying mechanism of ACS is plaque erosion (PE), which accounts for 25% of ACS cases [1]. Local hemodynamic flow plays a significant role in the development and progression of atherosclerosis [2]. Therefore, *in vivo* use of intravascular imaging to precisely differentiate atherosclerotic plaque morphology influenced by local hemodynamic flow is of increasing clinical interest.

Intravascular ultrasound (IVUS) and optical coherence tomography (OCT) are both diagnostic and guidance tools for interventional procedures, significantly contributing to our understanding of coronary artery disease [3]. IVUS has been the cornerstone of intracoronary imaging for >20 years and can provide information on plaque burden and remodeling. In contrast, OCT has advantages in detecting fibrous cap thickness and thrombus formation [4]. However, OCT has several limitations, such as the need for complete blood removal and the use of contrast agents, even in patients with chronic kidney disease; this sometimes precludes the assessment of the underlying mechanisms. Currently, case series support the potential value of high-definition IVUS (HD-IVUS) in evaluating PR and PE [5]. HD-IVUS, which uses 60 MHz transducers (Boston Scientific Corporation, 24235669, Marlborough, MA, USA), is the latest advancement in the development of this imaging technique, achieving better spatial resolution. In addition, 60 MHz HD-IVUS maintains a key potential advantage of IVUS over OCT—greater tissue penetration without requiring intraluminal blood removal [6]. However, the detection of PR and PE in clinical settings is primarily facilitated by OCT [7, 8]. Currently, no study has used 60 MHz HD-IVUS (Boston Scientific Corporation) for *in vivo* evaluation of atherosclerotic plaque morphology influenced by the local hemodynamic flow. This study examined the diagnostic performance of 60 MHz HD-IVUS in identifying plaque rupture and erosion, as well as thrombosis, using OCT-diagnosis as a standard reference in a rabbit model.

## 2. Materials and Methods

### 2.1 Animals

In this study, male New Zealand white (NZW) rabbits weighing 2.5–3.5 kg and 3–4 months old were purchased from the Animal Centre of Harbin Medical University. Surgery was performed in the Second Affiliated Hospital of the Harbin Medical University Laboratory. The rabbits were provided food and water ad libitum and individually housed in a common enriched environment 1 month before surgery.

### 2.2 Generation of a Rabbit Model of Atherosclerosis with Arterial Stenosis

**Fig. 1.**
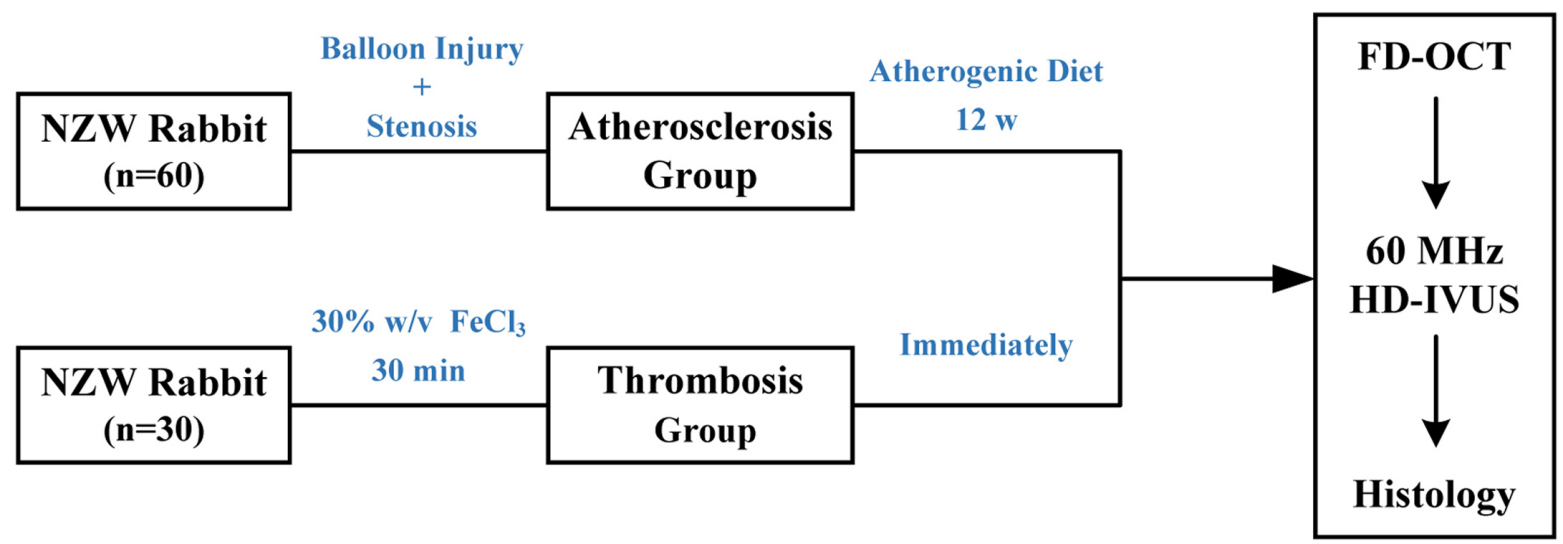
**Flow chart showing the animal groups and timelines of the experimental protocol *in vivo***. NZW, New Zealand white; HD-IVUS, high-definition intravascular ultrasound; FD-OCT, frequency-domain optical coherence tomography; w, week.

The protocol described below (Fig. 1) resulted from optimization through the establishment of a new rabbit model by our research group, which has the advantages of less trauma and a high survival rate. Surgery was performed in a sterile, controlled environment at room temperature (22–24 °C). According to the experimental requirements, 60 rabbits were used to establish a rabbit left common carotid artery (LCCA) stenosis model. Anesthesia was maintained during the experiment by intravenous infusion of ketamine that which was sufficient to abolish the corneal reflex.

After the NZW rabbits lost consciousness, we exposed the LCCA, as previously described [9]. The Emerge™ Monorail® Over-the-Wire PTCA Dilatation Catheter (2.50 × 20 mm; Boston Scientific Corporation, Marlborough, Massachusetts, USA) was inserted into the LCCA through the Samurai™ Straight Tip Guidewire (0.014 in × 190 cm, Boston Scientific Corporation, Marlborough, Massachusetts, USA). Since the ratio of the optimal diameter of balloon dilatation to the diameter of rabbit LCCA was 1.5:1, we inflated the catheter to 14 atm and retracted it three times, resulting in endothelial injury.

**Fig. 2.**
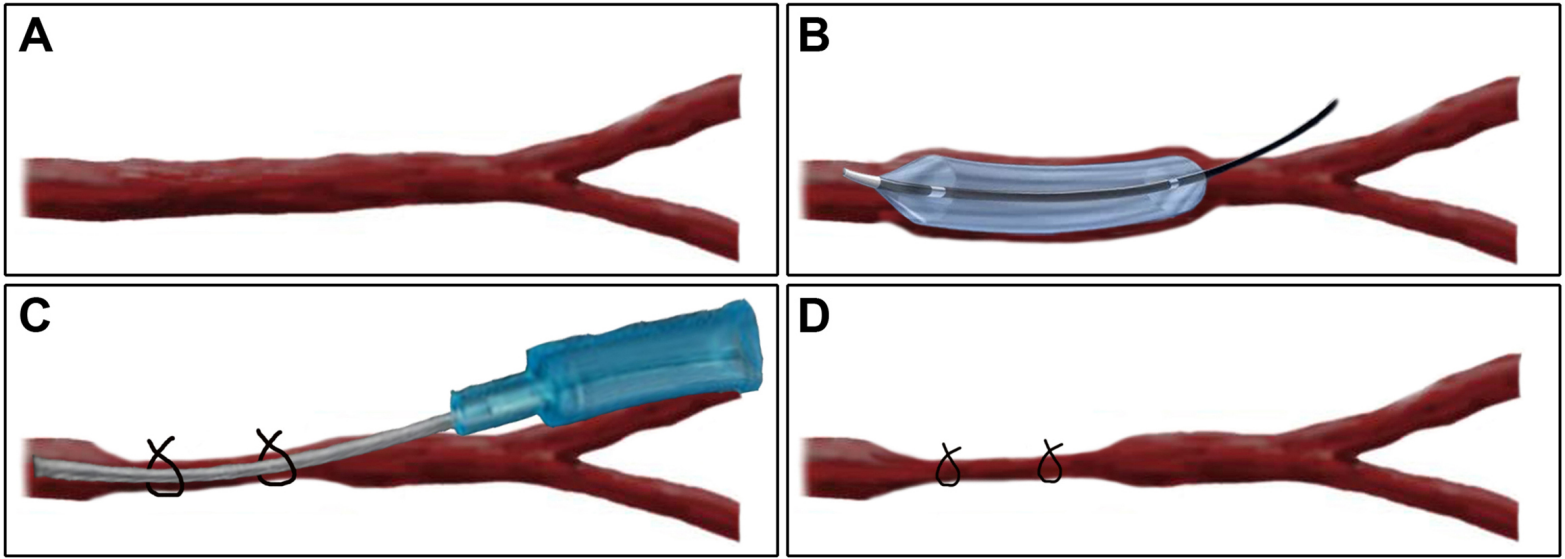
**Schematic representation of a combined arterial stenosis model after balloon injury of the LCCA in NZW rabbits**. (A) The LCCA was exposed. (B) Balloon injures endothelium. (C) The LCCA containing a 22-gauge plastic mandrel (outer diameter 0.6 mm) was tied with a nylon suture to cause arterial stenosis. (D) The plastic mandrel was then removed to restore blood flow, and the puncture site was sutured with 7-0 suture.

Local flow disturbance of the LCCA was induced by binding the LCCA to a plastic mandrel with nylon sutures (Fig. 2). The diameter of the rabbit LCCA was approximately 2 mm [10, 11]; therefore, we chose 22-gauge (o.d. 0.6 mm) needles to place in the LCCA, tying nylon sutures around the LCCA to form focal stenosis (lumen diameter reduction of approximately 70%). The plastic mandrel was then gently removed, leaving a ligature 0.6 mm of the LCCA diameter to resume blood flow. After all operations, the incision was closed with a 7-0 suture under aseptic conditions, and antibiotics were injected intravenously to prevent infection. The rabbits were then allowed to rest and were monitored daily for infection. After 12 weeks of an atherogenic diet (2% cholesterol and 6% peanut oil), all rabbits in our study had the LCCA exposed for OCT and 60 MHz HD-IVUS scanning, and then euthanized with a pentobarbital overdose.

### 2.3 Generating a Rabbit Thrombosis Model

**Fig. 3.**
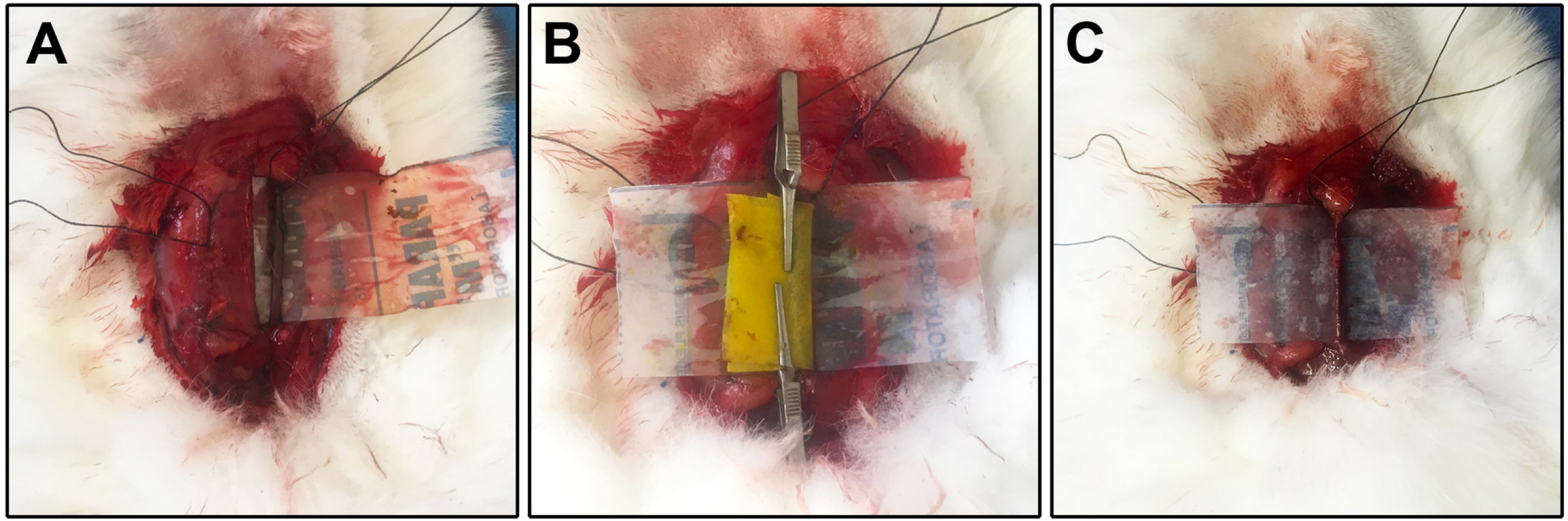
**Schematic representation of ${\mathrm{FeCl}}_{3}$ induced thrombosis model in rabbit LCCA**. (A) The LCCA was exposed and a piece of parafilm was placed underneath the LCCA. (B) ${\mathrm{FeCl}}_{3}$ injures the LCCA wall and exposes a thrombogenic surface in the lumen. (C) After 30 minutes, the residual ${\mathrm{FeCl}}_{3}$ was washed off with warm saline.

The ${\mathrm{FeCl}}_{3}$-induced arterial model of thrombosis is one of the most widely used animal models (Fig. 3). Thirty rabbits were randomly selected to establish the rabbit model of LCCA thrombosis. The ${\mathrm{FeCl}}_{3}$ injury model was established based on previously described procedures, with modifications to make it suitable for rabbits [12, 13]. We used the abovementioned steps to expose the LCCA. Thrombosis was then induced by applying a piece of filter paper (3 × 1.5 cm) saturated with 30% w/v ${\mathrm{FeCl}}_{3}$ wrapped around the adventitial surface of the LCCA. A piece of parafilm (4 × 2 cm) was placed underneath the LCCA to protect the surrounding tissue from injury. ${\mathrm{FeCl}}_{3}$ injures the vessel wall and exposes the thrombogenic surface of the lumen. The filter papers were removed after 30 min, followed by a washout of residual ${\mathrm{FeCl}}_{3}$ with warm saline. We scanned the arterial thrombus with OCT and 60 MHz HD-IVUS and euthanized each animal with a pentobarbital overdose.

### 2.4 Acquisition and Analysis of OCT Images

We inserted the imaging catheter into the LCCA and marked the vascular position at the head of the catheter as the starting point of the carotid segment, and the puncture port as the end point of the carotid segment. OCT and HD-IVUS were both used to scan the entire carotid segment. OCT imaging was performed using a Dragonfly™ Duo Imaging Catheter (LightLab Imaging Inc., Westford, MA, USA). After catheter placement, the OCT imaging catheter was pulled back over a longitudinal distance of up to 54 mm, at a rate of 20 mm/s using standalone electronic control of the pullback motor. The OCT analysis was performed using a dedicated offline review system. The minimum lumen area (MLA) and minimal lumen diameter (MLD) were determined for the culprit lesion. The reference lumen area (RLA) and reference lumen diameter (RLD) were set at a cross-section adjacent to the culprit lesion with the largest lumen and plaque burden of <50%. The percent area stenosis and diameter stenosis were calculated as follows: (RLA – MLA)/RLA × 100 and (RLD – MLD)/ RLD × 100, respectively.

In the rabbit model of atherosclerosis with arterial stenosis, OCT-atherosclerotic plaque was defined as loss of the normal “layered” appearance of the vessel wall with (i) increased intimal area, (ii) presence of highly reflective subintimal areas, and (iii) discontinuation of the internal elastic membrane [14]. OCT-PR was defined as fibrous cap disruption with a clear cavity formed inside the plaque; OCT-PE was defined as the presence of an attached thrombus overlying an intact and visible plaque, luminal surface irregularity at the culprit lesion in the absence of thrombus, or attenuation of underlying plaque by a thrombus without superficial lipids or calcification immediately proximal or distal to the thrombus site [15, 16]. In the rabbit thrombosis model, intracoronary thrombus was defined as a mass (diameter >250 mm) attached to the luminal surface or floating within the lumen, including red thrombus (red blood cell-rich), defined by high backscattering and attenuation, or white (platelet-rich) thrombus, defined by homogeneous backscattering with low attenuation [16, 17]. OCT-PR, OCT-PE, and OCT-thrombosis were independently evaluated by two experienced reviewers blinded to the 60 MHz HD-IVUS findings. In the event of a disagreement between the two reviewers, a third professional investigator intervened to reach a consensus.

### 2.5 Acquisition and Analysis of 60 MHz HD-IVUS Images

**Fig. 4.**
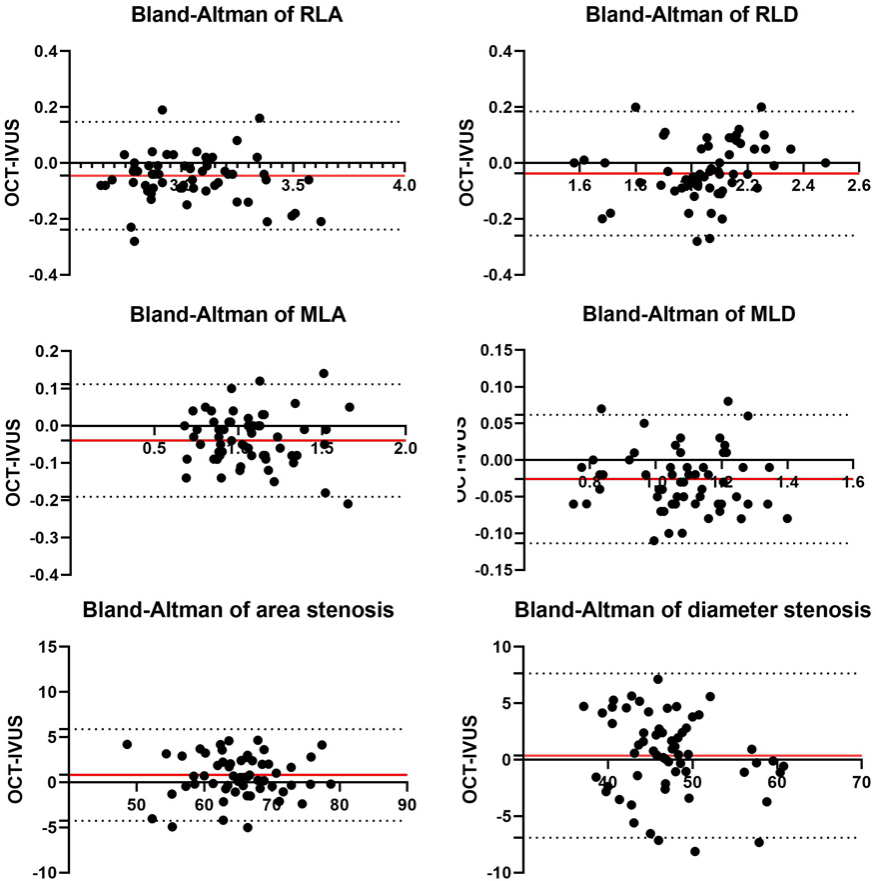
**Bland-Altman plots showing agreement of vessel measurements between OCT and 60 MHz HD-IVUS**. The red line represents the mean difference (bias); the grey dotted lines, 95% limits of agreement. IVUS, intravascular ultrasound; OCT, optical coherence tomography; RLA, reference lumen area; RLD, reference lumen diameter; MLA, minimum lumen area; MLD, minimal lumen diameter.

Following OCT imaging, 60 MHz HD-IVUS imaging was performed using OptiCross™ HD 60 MHZ coronary imaging catheters (Boston Scientific Corporation, Marlborough, MA, USA). The 60 MHz HD-IVUS catheter was advanced beyond the target lesion and automatically withdrawn at a pullback speed of 0.5 mm/s. 60 MHz HD-IVUS images were video recorded for subsequent analyses. 60 MHz HD-IVUS analysis was performed using offline Image Viewer software (Boston Scientific Image Viewer Software 04_29_2019_1, Boston Scientific Corporation, Marlborough, MA, USA). Plaque burden was defined as plaque and media cross-sectional area divided by an external elastic membrane cross-sectional area. HD-IVUS identification of an atherosclerotic lesion was defined as a segment with a 40% plaque burden in at least three consecutive frames [18]. MLA, MLD, RLA, and RLD were measured on a cross-section at the same location in OCT. To obtain matching HD-IVUS and FD-OCT images, we inserted the imaging catheter into the LCCA and marked the vascular position at the head of the catheter as the starting point of the carotid segment, and the puncture port as the end point of the carotid segment. OCT and HD-IVUS were both used to scan the entire carotid segment. HD-IVUS identification of PR was defined as a plaque cavity with an unfilled space within the plaque, beginning at the luminal-intimal border [19]. HD-IVUS identification of PE was defined as the presence of a normal vessel wall and minor intimal irregularities, with or without thrombus, suggesting the diagnosis of plaque erosion [20]. In contrast, in the presence of fibrotic or lipid plaques, the finding of surface irregularities or layered images without cap rupture is also suggestive of plaque erosion [20]. HD-IVUS differentiated between red and white thrombi as follows: the average energy ratio backscattered by a red thrombus was constant with increasing tissue depth; conversely, it attenuated for white thrombi. Red thrombi were less homogeneous and had more backscatter compared to white thrombi [21]. Two experienced observers blinded to the OCT findings analyzed the 60 MHz HD-IVUS images. In the event of a disagreement between the two reviewers, a third professional investigator intervened to reach a consensus. We assessed the vessel measurement agreement between OCT and 60 MHz HD-IVUS using Bland-Altman plots (Fig. 4).

### 2.6 Statistical Analysis

Statistical analysis was performed using SPSS version 23 (IBM, Armonk, USA). Quantitative variables are presented as mean ± standard deviations, and qualitative variables are presented as total numbers and percentages. An independent two-sample *t*-test and Bland-Altman plots were used to assess the differences between the two data sets. Statistical significance was defined as a two-sided *p*-value of <0.05. With plaque morphology and thrombosis identified by OCT as the gold standard, the sensitivity, specificity, positive predictive value (PPV), negative predictive value (NPV), and accuracy of 60 MHz HD-IVUS were calculated.

## 3. Results

### 3.1 Quantitative Measurements by 60 MHz HD-IVUS and OCT

**Fig. 5.**
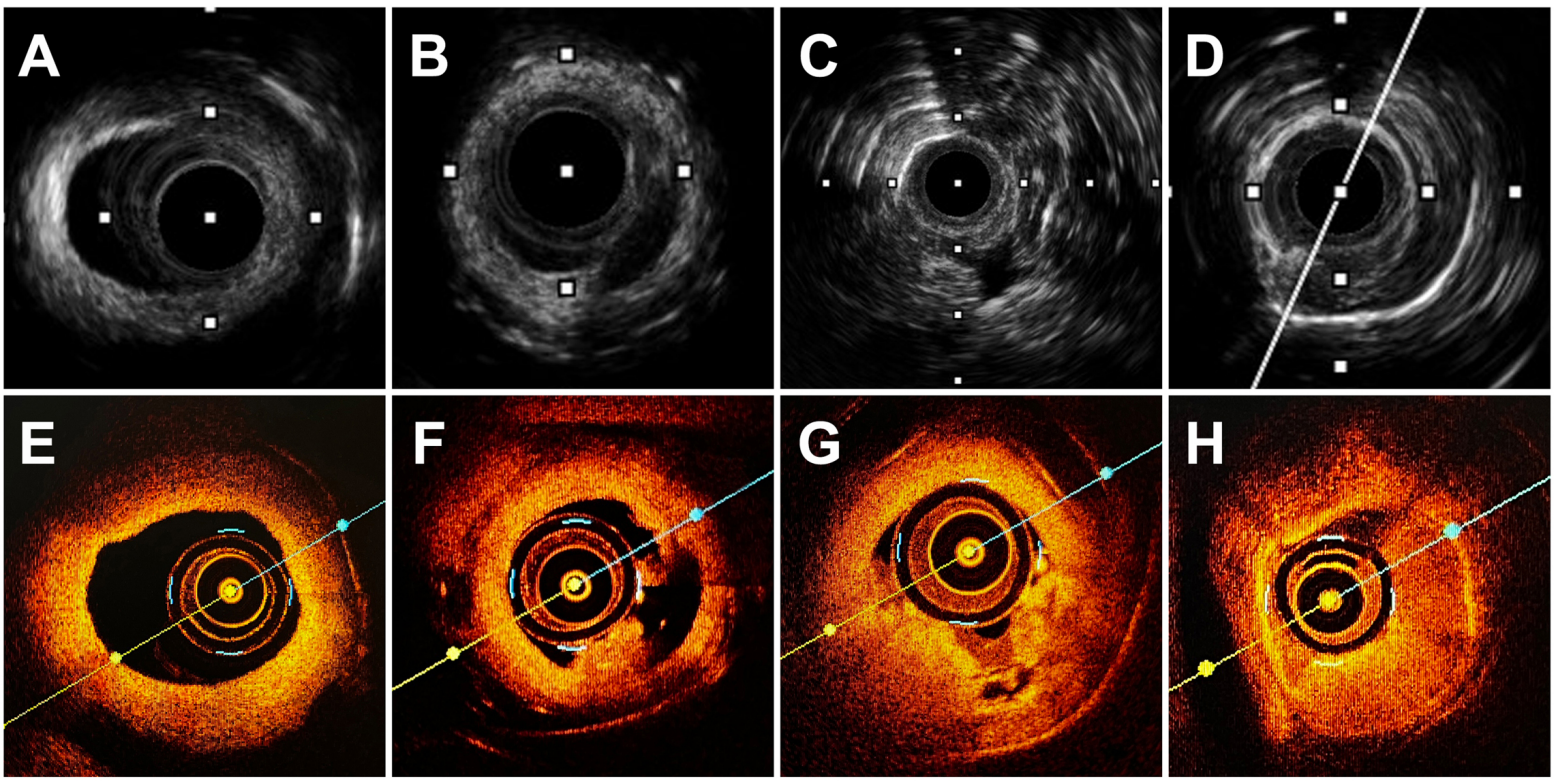
**Coregistered 60 MHz HD-IVUS and OCT images of rabbit carotid arteries**. 60 MHz HD-IVUS images are displayed in the upper panels, and OCT images are displayed the lower panels. (A,E) Imaging of atherosclerotic plaque observed with both modalities. (F) OCT demonstrates PR characterized by a fibrous-cap disruption and a cavity formation inside the plaque. (B) HD-IVUS reveals a cavity inside the plaque. (G) OCT revealed a small thrombus without signs of plaque rupture, fulfilling the criteria of definitive PE. (C) HD-IVUS revealed a minor intimal irregularities with a small thrombus, also suggestive of PE. (D,H) Imaging of thrombosis observed with both modalities.

We established 60 rabbit models of LCCA balloon injury combined with arterial stenosis. At the end of the third month after surgery, we performed both OCT and HD-IVUS scans of the LCCA (Fig. 5). The quantitative measurements using HD-IVUS and OCT are summarized in Table 1. There were no significant differences between HD-IVUS and OCT regarding RLA (3.05 ± 0.25 vs. 3.01 ± 0.23 ${\mathrm{mm}}^{2}$; *p* = 0.3125) and RLD (2.06 ± 0.17 vs. 2.03 ± 0.18 mm; *p* = 0.2546), or in the assessment of MLA (1.08 ± 0.25 vs. 1.04 ± 0.24 ${\mathrm{mm}}^{2}$; *p* = 0.3841) and MLD (1.09 ± 0.15 vs. 1.06 ± 0.14 mm; *p* = 0.3439).

**Table 1.** **Vessel Measurements by 60 MHz HD-IVUS and OCT**.

	HD-IVUS (n = 60)	OCT (n = 60)	*p* value
RLA, ${\mathrm{mm}}^{2}$	3.05 ± 0.25	3.01 ± 0.23	0.3125
RLD, mm	2.06 ± 0.17	2.03 ± 0.18	0.2546
MLA, ${\mathrm{mm}}^{2}$	1.08 ± 0.25	1.04 ± 0.24	0.3841
MLD, mm	1.09 ± 0.15	1.06 ± 0.14	0.3439
area stenosis, %	64.70 ± 6.28	65.52 ± 6.14	0.4736
diameter stenosis, %	46.94 ± 6.18	47.30 ± 5.58	0.7362

Data are presented as mean ± SD. HD-IVUS, high-definition intravascular ultrasound; OCT, optical coherence tomography; RLA, reference lumen area; RLD, reference lumen diameter; MLA, minimum lumen area; MLD, minimal lumen diameter.

### 3.2 Diagnostic Performance of 60 MHz HD-IVUS for OCT-Defined Atherosclerotic Plaque Identification

We used OCT-defined atherosclerotic plaques and identified plaque morphological characteristics as the gold standard to assess the sensitivity, specificity, PPV, NPV, and accuracy of HD-IVUS for identifying lesions. In the 60 rabbit LCCA balloon injury combined with the arterial stenosis model, 52 atherosclerotic plaques were identified using OCT, and 50 atherosclerotic plaques were identified using HD-IVUS. Of the 52 OCT-defined atherosclerotic plaques, HD-IVUS identified 48 atherosclerotic plaques, and four had no atherosclerotic plaques (Table 2a). Of the remaining eight models in which OCT did not identify atherosclerotic plaques, HD-IVUS identified no atherosclerotic plaques in six LCCA cases and two atherosclerotic plaque formation cases (Table 2a). These results suggest that the sensitivity, specificity, PPV, NPV, and accuracy of HD-IVUS in identifying OCT-defined atherosclerotic plaques were 92.3, 75.0, 96.0, 60.0, and 90.0%, respectively (Table 2b).

**Table 2a.** **Assessment of 60 MHz HD-IVUS for OCT-defined atherosclerotic plaque**.

	OCT (n = 60)		Total
	Atherosclerotic Plaque (+)	Atherosclerotic Plaque (–)	
HD-IVUS (n = 60)			
Atherosclerotic Plaque (+)	48	2	50
Atherosclerotic Plaque (–)	4	6	10
	52	8	60

HD-IVUS, high-definition intravascular ultrasound; OCT, optical coherence tomography.

**Table 2b.** **Diagnostic Performance of 60 MHz HD-IVUS**.

Sensitivity (%)	92.3
Specificity (%)	75.0
PPV (%)	96.0
NPV (%)	60.0
Accuracy (%)	90.0

HD-IVUS, high-definition intravascular ultrasound; PPV, positive predictive value; NPV, negative predictive value.

### 3.3 Diagnostic Performance of 60 MHz HD-IVUS for OCT-Defined PR Identification

In 60 LCCA balloon injury rabbit models with arterial stenosis, 25 PRs were identified using OCT and 24 using HD-IVUS. Therefore, HD-IVUS identified 23/25 (92.0%) OCT-defined PRs (Table 3a). We randomly selected 10/35 cases in which PR was not identified by OCT as the control group. Of these 10 cases, there were nine (90.0%) in which PR was not identified by HD-IVUS (Table 3a). Using OCT-defined PR as the gold standard, the sensitivity, specificity, PPV, NPV, and accuracy of HD-IVUS in identifying PR were 92.0, 90.0, 95.8, 81.8, and 91.4%, respectively (Table 3b).

**Table 3a.** **Assessment of 60 MHz HD-IVUS for OCT-Defined PR**.

	OCT		Total
	Plaque Rupture (+)	Plaque Rupture (–)	
HD-IVUS			
Plaque Rupture (+)	23	1	24
Plaque Rupture (–)	2	9	11
	25	10	35

HD-IVUS, high-definition intravascular ultrasound; OCT, optical coherence tomography.

**Table 3b.** **Diagnostic Performance of 60 MHz HD-IVUS**.

Sensitivity (%)	92.0
Specificity (%)	90.0
PPV (%)	95.8
NPV (%)	81.8
Accuracy (%)	91.4

HD-IVUS, high-definition intravascular ultrasound; PPV, positive predictive value; NPV, negative predictive value.

### 3.4 Diagnostic Performance of 60 MHz HD-IVUS for OCT-Defined PE Identification

Consistent with the above steps, OCT and HD-IVUS identified 10 and 11 PE cases, respectively. Of the 10 PEs identified by OCT, HD-IVUS identified 8 PEs (80.0%), while the remaining two were not found (Table 4a). However, of the 10 cases in which PE was not identified by OCT, HD-IVUS identified no PE in seven cases (70.0%) and PE in three cases (Table 4a). These above results indicate that the sensitivity, specificity, PPV, NPV, and accuracy of HD-IVUS in identifying OCT-defined PE were 80.0, 70.0, 72.7, 77.8, and 75.0%, respectively (Table 4b).

**Table 4a.** **Assessment of 60 MHz HD-IVUS for OCT-Defined PE**.

	OCT		Total
	Plaque Erosion (+)	Plaque Erosion (–)	
HD-IVUS			
Plaque Erosion (+)	8	3	11
Plaque Erosion (–)	2	7	9
	10	10	20

HD-IVUS, high-definition intravascular ultrasound; OCT, optical coherence tomography.

**Table 4b.** **Diagnostic Performance of 60 MHz HD-IVUS**.

Sensitivity (%)	80.0
Specificity (%)	70.0
PPV (%)	72.7
NPV (%)	77.8
Accuracy (%)	75.0

HD-IVUS, high-definition intravascular ultrasound; PPV, positive predictive value; NPV, negative predictive value.

### 3.5 Diagnostic Performance of 60 MHz HD-IVUS for OCT-Defined Thrombosis Identification

ACS is caused by coronary thrombosis, secondary to PR and PE. Therefore, we established a rabbit LCCA thrombosis model and performed OCT and HD-IVUS scanning of the LCCA immediately after the surgery. Of the 30 LCCA thrombus models, 25 were identified by OCT and 23 by HD-IVUS. HD-IVUS identified 22/25 LCCA thrombi defined by OCT (88.0%), while the remaining three were not found (Table 5a). In addition, among the LCCA cases in which OCT identified no thrombi, HD-IVUS identified four cases without LCCA thrombosis (80.0%) and one case with LCCA thrombosis (Table 5a). Based on these results, the sensitivity, specificity, PPV, NPV, and accuracy of HD-IVUS for identifying OCT-defined thrombosis were 88.0, 80.0, 95.7, 57.1, and 86.7%, respectively (Table 5b).

**Table 5a.** **Assessment of 60 MHz HD-IVUS for OCT-Defined Thrombosis**.

	OCT (n = 30)		Total
	Thrombosis (+)	Thrombosis (–)	
HD-IVUS (n = 30)			
Thrombosis (+)	22	1	23
Thrombosis (–)	3	4	7
	25	5	30

HD-IVUS, high-definition intravascular ultrasound; OCT, optical coherence tomography.

**Table 5b.** **Diagnostic Performance of 60 MHz HD-IVUS**.

Sensitivity (%)	88.0
Specificity (%)	80.0
PPV (%)	95.7
NPV (%)	57.1
Accuracy (%)	86.7

HD-IVUS, high-definition intravascular ultrasound; PPV, positive predictive value; NPV, negative predictive value.

## 4. Discussion

This study is the first to show a direct comparison of atherosclerotic plaque morphology between 60 MHz HD-IVUS and OCT images. The main findings of our study were the following: (1) in the rabbit model of atherosclerosis with arterial stenosis, the diagnostic performance of 60 MHz HD-IVUS showed sensitivity and specificity of 92.0 and 90.0% for identifying OCT-defined PR and 80.0 and 70.0% for OCT-defined PE, respectively; (2) in the rabbit carotid thrombosis model, 60 MHz HD-IVUS showed a high sensitivity (88.0%) and specificity (80.0%) for identifying OCT-defined thrombosis.

Currently, precise *in vivo* differentiation between PR and PE in culprit lesions of ACS is a significant challenge for intravascular imaging. Both OCT and HD-IVUS are currently the most commonly used intravascular imaging techniques for PR and PE identification in patients with ACS [22]. OCT uses infrared light, which confers high spatial resolution in the 15–20 μm range, and remains highly consistent with pathology [23, 24]. However, OCT has some limitations, such as shallow scanning depth, need for complete blood removal, use of contrast media, and poor ability to visualize post-thrombotic structures [25]. Previous IVUS studies have demonstrated that IVUS has important clinical implications in assessing coronary plaque morphology but may be limited in detecting PR and PE due to a lower spatial resolution [26]. To achieve better spatial resolution, a 60 MHz HD-IVUS transducer (Boston Scientific Corporation, Marlborough, MA, USA) was successfully designed. 60 MHz HD-IVUS has evolved into a new generation of IVUS imaging technology, with an axial resolution of approximately 20–40 μm, faster catheter pullback speeds up to 10 mm/s, and rapid image acquisition of 60 frames/s, while maintaining the potential benefit of IVUS over OCT: namely, its tissue penetration and image acquisition not requiring contrast injection [6]. We measured the cross-section of the LCCA lumen in 60 rabbit models, and the results showed that the minimum lumen area and diameter measured by 60 MHz HD-IVUS and OCT were not significantly different, which is inconsistent with the results of previous studies [27, 28]. In our study, HD-IVUS showed an excellent concordance with OCT on LCCA measurements. Previous studies have shown that the lumen area (LA) measured by OCT is similar to the actual phantom LA, whereas IVUS overestimates the LA [27]. A potential reason for this discrepancy may be that the resolution of conventional IVUS is lower than that of OCT [29]. Our study confirms that the novel 60 MHz HD-IVUS with better axial resolution can minimize these differences.

60 MHz HD-IVUS facilitates the analysis of the luminal surface; therefore, it can be used to detect PR and PE. Local hemodynamic alterations may shift stable plaques towards vulnerable plaques by affecting endothelial function and local inflammation [30]. Therefore, local hemodynamic disruption are essential for the development of PR and PE. We modified the previous rabbit atherosclerosis model establishment method to induce local stenosis after balloon injury of the carotid artery. Our results showed that 60 MHz HD-IVUS identified 24 PRs and 11 PEs in 52 OCT-defined atherosclerotic plaques at the end of the third postoperative month. Our study indirectly confirmed that local hemodynamic alterations are among the most important factors leading to the development of ACS. A calcified nodule is one of the most common causes of ACS [16]. In the rabbit model of this study, no obvious calcified nodules were found. It is possible that the high fat atherogenic diet may have been fed for too short a time and failed to form vascular calcification. This is consistent with previous studies on animal models where vascular calcification often required genetically modified models or substance applications to induce disease in a reasonably short timeframe [31].

Compared with conventional IVUS studies, IVUS of the coronary arteries might be preferably imaged at 60 MHz than at 40 MHz [32]. In a previous study comparing IVUS and OCT, 20 MHz IVUS could only identify 23.6% of OCT-defined PR [33]. Another study comparing 40 MHz IVUS and OCT in patients with ACS showed a 40 and 73% incidence of PR identified by IVUS and OCT, respectively [34]. Conventional IVUS cannot determine the presence or absence of small ruptures owing to its coarse resolution; hence why this technique poorly identifies PE [35]. However, in patients with ascertained PE, OCT can be visualized using HD-IVUS [5]. Recently, near-infrared spectrum (NIRS)-IVUS has been shown to have a sensitivity and specificity of 97% and 96%, respectively, for identifying OCT-PR [15]. Another study showed that HD-IVUS had high sensitivity (84.8%), but modest specificity (57.1%) for identifying OCT-derived PE [36]. In the present study, 60 MHz HD-IVUS showed high sensitivity (92.0%) and specificity (90.0%) for identifying OCT-defined PR but modest sensitivity (80.0%) and specificity (70.0%) for identifying OCT-defined PE. It must be recognized that the application of HD-IVUS to diagnose PE remains challenging. However, when patients with coronary heart disease have concomitant renal dysfunction, the application of HD-IVUS in cases with suspected PE can minimize the risk of contrast-induced nephropathy.

Conventional IVUS examination often poorly visualizes thrombosis [37]. 60 MHz HD-IVUS may overcome the limitations of conventional IVUS and offer more reliable thrombus detection. As described in our case, we established a rabbit thrombus model with acute ${\mathrm{FeCl}}_{3}$-induced LCCA thrombosis. The results showed that 60 MHz HD-IVUS had high sensitivity and specificity for identifying OCT-defined thrombosis in our model. However, OCT still provided better visualization of thrombus contour compared to HD-IVUS. We believe that this is the first report of 60 MHz HD-IVUS and OCT for direct comparison of acute thrombosis.

A recent study comparing HD-IVUS and OCT to coregistered native coronary arteries has shown that superior tissue penetration of HD-IVUS allows complete vascular evaluation through fibrous and lipidic plaques, while the OCT signal is strongly attenuated [38]. When compared with OCT, HD-IVUS also has the advantage of procedural simplicity. HD-IVUS can provide clear images of plaque morphology without blood removal, even among operators who are not familiar with the imaging procedures [39]. These results suggest that 60 MHz HD-IVUS may be an alternative modality for the OCT-guided pathogenesis of ACS.

First, the primary limitation of this study is that we used atherosclerotic plaques, PR, PE, and thrombosis diagnosed by OCT as reference standards. Second, 60 rabbit atherosclerosis models and 30 rabbit thrombosis models were established for intravascular imaging studies of plaque morphology and thrombosis, respectively; therefore, selection bias may be possible when considering the number of samples, and larger prospective studies are required to validate these results. Third, although meticulous care was taken to avoid excessive mechanical trauma, repeated manipulation of the imaging catheter may still have induced iatrogenic fibrous cap disruption, affecting the identification of PR by other modalities. Fourth, in addition to PR and PE, there are other ACS mechanisms such as calcified nodules that we did not assess [1]. However, the frequency of these mechanisms was low. The precise diagnostic performance of 60 MHz HD-IVUS in identifying PR and PE might allow for selecting appropriate therapies specific to unstable lesion types in ACS.

## 5. Conclusions

60 MHz HD-IVUS can be used to identify PR and thrombosis accurately. Further studies are required to confirm the clinical value of this novel imaging technique in the diagnosis of PE.

## Data Availability

All data generated or analyzed during this study are included in this published article.
